# The Influence of Maturity Status on Anthropometric Profile and Body Composition of Youth Goalkeepers

**DOI:** 10.3390/ijerph17218247

**Published:** 2020-11-08

**Authors:** Andrea Di Credico, Giulia Gaggi, Barbara Ghinassi, Gabriele Mascherini, Cristian Petri, Riccardo Di Giminiani, Angela Di Baldassarre, Pascal Izzicupo

**Affiliations:** 1 Department of Medicine and Aging Sciences, University “G. D’Annunzio” of Chieti-Pescara, 66100 Chieti, Italy; andrea.dicredico@unich.it (A.D.C.); giulia.gaggi@unich.it (G.G.); b.ghinassi@unich.it (B.G.); izzicupo@unich.it (P.I.); 2 Department of Experimental and Clinical Medicine, University of Florence, 50121 Firenze, Italy; gabriele.mascherini@unifi.it (G.M.); cristian.petri@unifi.it (C.P.); 3 Department of Biotechnological and Applied Clinical Sciences, University of L’Aquila, 67100 L’Aquila, Italy; riccardo.digiminiani@univaq.it

**Keywords:** anthropometry, somatic maturation, body composition, cardiometabolic risk, youth athletes, soccer, obesity, physical health

## Abstract

The anthropometric profile assessment is an important aspect to consider during the growth stages of youth sport practitioners due to its usefulness in controlling maturity status and overall health. We performed an anthropometric profile evaluation in a sample of youth goalkeepers (*n* = 42) during a training camp, dividing them into three categories based on their years from peak height velocity (YPHV). We also checked if the selection of goalkeepers was associated with the birth quartile. The results showed that most of the participants’ anthropometric parameters followed the normal trend according to the maturation stages. However, several subjects showed an overweight/obese condition and/or high waist circumference. Non-optimal values were found, mostly in the group of goalkeepers around the PHV. In addition, no selection based on birth quartile was seen. Therefore, the anthropometric profile and body composition of youth goalkeepers are physiologically affected by maturity status. However, several subjects were found to be overweight/obese and at cardiometabolic risk, suggesting that children and adolescents, although practicing sport, should pay attention to potentially contributing factors such as the attainment of the recommended levels of physical activity, lowering sedentary time, and adopt a healthy lifestyle.

## 1. Introduction

Anthropometry is a valuable technique for assessing the size, proportions, and composition of the human body [1]. Common anthropometric measurements include height, weight, skinfolds, and circumferences, frequently used as nutritional status indexes, health, and growth [2]. In particular, anthropometry and the derived body composition parameters are useful tools to evaluate both the children and adolescents at the different stages of growth [3]. In this regard, Mirwald et al. developed a formula to obtain the peak height velocity (PHV) [4]. PHV is defined as the maximum velocity of growth in stature or the growth spurt in height, and it represents a valid indicator of somatic maturity status [3]. Moreover, years from PHV (YPHV) are obtained subtracting the age of PHV from chronological age, and characterize a non-invasive method to assess the trend of maturity in children [4].

The anthropometric profile, body composition, and maturity status are parameters of great importance in sports [5,6,7]. For example, improved body composition in athletes is associated with greater strength [8] and cardiorespiratory fitness [9]. Furthermore, anthropometric variables and maturity are associated with team sports success, such as soccer [10]. For instance, in different soccer categories based on age groups, players in advanced maturation tend to be taller and heavier than others [11,12]. Furthermore, specific anthropometric characteristics represent a prerequisite to playing in different positions considering the different roles (e.g., defenders, forwards, and goalkeepers) [13].

Specifically, goalkeepers tend to be taller and heavier due to the specific requirements of their role. Thus soccer teams are inclined to select them, taking into account such anthropometric characteristics [14]. Accordingly, it seems that coaches and athletic trainers select youth athletes based on their date of birth, preferring those who are more advanced in growth, especially in high-level teams [15]. This phenomenon is known as the relative age effect (RAE), a condition in which the relative birth quarter distribution in a sample of athletes is not evenly distributed [16]. Indeed, in such a situation, the older athletes, born close to the beginning of the year, represent the majority of the team [17]. In contrast, these preferential choices are not evident within soccer teams of lower levels [18]. 

However, with the increasing adoption of unhealthy lifestyles (including the massive usage of electronic devices and social media), children and adolescents can spend a large amount of time in sedentary behaviors and fail to reach the recommended physical activity levels [19,20], despite sports participation [21]. Sedentary behaviors, physical inactivity, and incorrect nutritional habits are detrimental factors for overall health [22] and are responsible for increasing all-cause mortality [23] at all population levels [24]. Thus, examinations indicating pathological states such as obesity or cardiometabolic risk in children and adolescents participating in sport are valuable procedures [25]. In this regard, different parameters can be assessed. For example, body mass index (BMI) is extensively used to recognize subjects having excessive adiposity [4,26,27,28]. However, BMI usage has significant limitations, as it does not allow discrimination between fat mass (FM) and free fat mass (FFM) [27]. Thus, body FM and the percentage of fat mass (%FM) are usually assessed to overcome BMI limits. However, even the %FM alone does consider factors such as height and FFM [29]. In this regard, the fat mass index (FMI) and fat-free mass index (FFMI) are two useful estimates to use along with BMI, absolute FM, and %FM for assessing body composition using anthropometry [30].

Other valuable methods are skinfold and circumference measurements and the ratios between various anthropometric parameters [28,31,32]. Notably, the skinfold thickness is a valid anthropometric indicator of fatness because it is widely representative of adipose tissue [33]. Moreover, higher waist circumferences are associated with an increased health risk, and measures such as waist/height ratio represent a useful means to stratify cardiometabolic risk in the youth population, taking into account sex and age [34,35]. Given the importance of such measures, various references for children and adolescents exist [31,36]. Hence, performing periodic anthropometric assessments is of utmost importance in children and adolescents, particularly in a team sport context, where such analysis provides insight about the ideal predisposition for a specific role/position, performance, and health as well [37]. In particular, the assessment of maturity status allows considerations beyond the simple chronological age, body size, proportion, and composition.

Therefore, this observational study aimed to investigate how maturity status, based on YPHV, can influence the anthropometric profile and body composition of youth goalkeepers from different soccer teams, indicating the differences in the different stages of maturation. We also analyzed how the different anthropometric variables correlated with YPHV. Finally, we checked if maturity status could lead to the presence of RAE in the different groups.

## 2. Materials and Methods

### 2.1. Participants

Forty-two young male goalkeepers were recruited from different soccer teams during a training camp dedicated to youth goalkeepers. Participants were classified into three groups: pre-PHV (*n* = 12, age = 11.98 ± 0.93), circa-PHV (*n* = 14, age = 13.74 ± 0.74) and post-PHV (*n* = 16, age = 15.99 ± 0.72). All participants were involved in soccer as goalkeepers from at least one year and performed 3 to 5 training sessions per week. The study was conducted in accordance with the Declaration of Helsinki. This study is part of a project of the Tuscany Region called “Sports Medicine to support regional surveillance systems”; it was approved by the Regional Prevention Plan 2014–2018 with the code O-Range18. Informed consent was obtained from all the participants before inclusion in the study.

### 2.2. Anthropometry and Body Composition

All the anthropometric measurements were performed by a certified specialist (i.e., a level 1 certification of the International Society for the Advancement of Kinanthropometry (ISAK)). Subjects wore light clothing and had fasted for at least 12 h before the assessments. Height was measured to the nearest 0.1 cm, and body weight was measured to the nearest 0.1 kg using a stadiometer with a balance-beam scale (Seca 200, Seca, Hamburg, Germany). Triceps and subscapular skinfolds were measured using a Cescorf skinfold caliper (Cescorf, Porto Alegre, Brazil). Arm span, waist circumferences (WC), and mid-arm circumference (MAC) were measured using a Cescorf anthropometric tape (Cescorf, Porto Alegre, Brazil). The percentage of fat-mass was calculated according to Slaughter [38] as follows:

(1) $$Prepubescent\;White\;Males:\;\%FM=1.21\;\left(triceps\;+\;subscapular\right)\;-\;0.008\;{\left(triceps+subscapular\right)}^{2}-\;1.7$$

(2) $$\mathrm{Pubescent}\mathrm{White}\mathrm{Males}:\;\%\mathrm{FM}=1.21(triceps\;+\;subscapular)-\;0.008\;{\left(triceps+subscapular\right)}^{2}-\;3.4$$

(3) $$\mathrm{Postpubescent}\mathrm{White}\mathrm{Males}:\;\%\mathrm{FM}=1.21(triceps\;+\;subscapular)-\;0.008\;{\left(triceps+subscapular\right)}^{2}-\;5.5$$

*For sum of triceps and subscapular greater than* 35 mm: *%FM* = 0.783 *(triceps* + *subscapular)* + 1.6 (4)

Mid-arm muscle circumference (MAMC), mid-arm area (MAA), mid-arm muscle area (MAMA), mid-arm fat area (MAFA), and arm fat index (AFI) were calculated as follows:

(5) $$MAMC=MAC-\left(\pi \;\times \;\frac{Triceps\;skinfold}{10}\right)$$

(6) $$MAA=\frac{MAC^{2}}{4\;\times \;\pi }$$

(7) $$MAMA=\frac{MAMC^{2}}{4\;\times \;\pi }$$

(8) MAFA=MAA−MAMA

(9) $$AFI=100\;\times \;\frac{MAFA}{MAA}$$

Body mass index (BMI) was calculated as weight in kilograms divided by the square of height, expressed in meters. Participants who had a WC >90th percentile, considering McCarthy’s waist circumference as a reference [39], were considered to have abdominal obesity, and a cutoff of 0.5 was used to differentiate low waist to height ratio (W/Hr) from high W/Hr [28,40]. 

### 2.3. Age and Maturity Status

According to the estimated age at PHV, the maturity status was defined based on the Mirwald et al. equation [4]. Participants were classified into three groups based on their YPHV: pre-PHV (offset <−1 years), circa-PHV (≤±1 years), and post-PHV (offset >+1 years), as a previous study reported [41].

### 2.4. Statistical Analysis

The Shapiro–Wilk test was performed to check the normality of the data. The analysis of variance (ANOVA) was used to determine the differences between maturity status group (defined as fixed factor) in body composition and anthropometric parameters (defined as dependent variables), and partial eta squared (η^2^_p_) was calculated to indicate the effect-size (small = 0.01, medium = 0.06, large = 0.14) [42]. When the analysis of variance (ANOVA) showed significant results, a Tukey’s post hoc test was used to confirm where the differences occurred. Pearson’s correlation coefficient was used to determine the extent of correlation between YPHV and anthropometric measures. Following the appropriate indications [43], the magnitude of correlations was considered as: *r* = 0.00–0.09, negligible; *r* = 0.10–0.39, weak; *r* = 0.40–0.69, moderate; *r* = 0.70–0.89, strong; *r* = 0.90–1.00, very strong. Finally, when the necessary conditions were met, the chi-square (χ^2^) test for independence was used to check the association between maturity status and the selected categories (BMI, waist circumference, birth quartile, growth velocity, and achievement of peak growth). When data were not normally distributed, the Kruskal–Wallis with Dunn’s post-hoc test was used instead of ANOVA and Tukey’s post hoc, and Spearman’s Rho was used instead of Pearson’s r. Descriptive data are presented as mean ± standard deviation, while categorical data as frequency (count and percentage of the group, total count, and percentage of the total). The statistical significance was set at <0.05. 

## 3. Results

### 3.1. Anthropometric Profile

Table 1 shows the descriptive statistics for the participants’ anthropometric and body composition variables divided by maturity offset. 

Age, height and weight were significantly different between groups [*p* < 0.001; F (2,39) = 54.732, *p* < 0.001, η^2^_p_ = 0.737; F (2,39) = 23.945, *p* < 0.001, η^2^_p_ = 0.551, respectively], however post hoc analysis did not shows differences between circa-PHV and post-PHV regarding weight (62.66 ± 10.17 vs. 69.29 ± 10.51, *p* = 0.163). Similarly, statistical difference was found for sitting height [F (2,39) = 54.824, *p* < 0.001, η^2^_p_ = 0.744] and arm span [F (2,39) = 34.722, *p* < 0.001, η^2^_p_ = 0.640]. On the other hand, both arm span to height ratio (AS/Hr) and sitting height to height ratio (SH/Hr) were similar in the three groups (*p* = 0.522; *p* = 0.330, respectively). 

Regarding skinfolds measurements, a significantly difference was seen for triceps skinfold (*p* = 0.042) but not for subscapular skinfold, (*p* = 0.143) with circa-PHV showing the highest values. The mid-arm circumference (MAC) was statistically different [F (2,39) = 7.393, *p* = 0.002, η^2^_p_ = 0.275] although the Tukey’s post hoc demonstrates that such a variable was similar between circa-PHV and post-PHV (26.89 ± 4.31 vs. 28.30 ± 2.03, *p* = 0.454). Furthermore, the derived mid-arm muscle circumference (MAMC) showed even more significant difference (*p* < 0.001) and in this case the post hoc demonstrates differences also between circa-PHV and post-PHV (21.99 ± 3.70 vs. 25.17 ± 1.57, *p* = 0.005). Similarly, both the mid-arm area (MAA) and the mid-arm muscle area (MAMA) were significantly different between groups [F (2,39) = 7.610, *p* = 0.002, η^2^_p_ = 0.281; F (2,39) = 23.213, *p* < 0.001, η^2^_p_ = 0.543], respectively. However the MAA was similar when comparing the circa-PHV with the post-PHV (58.96 ± 17.20 vs. 64.07 ± 9.54, *p* = 0.527). In addition, also the arm fat index (AFI) showed a significant difference between the three groups of goalkeepers (*p* = 0.005), but with pre-PHV and circa-PHV participants showing similar results. On the other hand, mid-arm fat area (MAFA) did not showed differences (*p* = 0.168). 

The WC was significantly different between the groups [F (2,39) = 7.782, *p* = 0.001, η^2^_p_ = 0.285], however the post hoc test showed no difference between circa-PHV and post-PHV (75.21 ± 6.96 vs. 74.09 ± 4.47, *p* = 0.869). Also waist to height ratio (W/Hr) was different between the groups (*p* = 0.029). Finally, the percentage of predicted adult height (%PAH) was significantly different (*p* < 0.001), while the predicted adult height (PAH) showed no significant differences [F (2,39) = 0.854, *p* = 0.434, η^2^_p_ = 0.042].

### 3.2. Body Composition

The three groups of goalkeepers showed a statistically different BMI [F (2,39) = 3.798, *p* = 0.031, η^2^_p_ = 0.163] although the values between circa-PHV and post-PHV were comparable (22.57 ± 2.69 vs. 22.06 ± 2.64, *p* = 0.883). Similarly, absolute fat mass was different [F (2,39) = 8.173, *p* = 0.001, η^2^_p_ = 0.295] while the post hoc revealed that circa-PHV and post-PHV participants fat mass was similar (10.93 ± 2.22 vs. 10.33 ± 1.76, *p* = 0.649). The %FM was also significantly different (*p* < 0.001) and showed a decreasing trend from pre-PHV to post-PHV. Likewise, the fat mass index (FMI) and the fat free mass index (FFMI) were different between groups [*p* = 0.016; F (2,39) = 5.994, *p* = 0.005, η^2^_p_ = 0.235, respectively], with a significant difference between circa-PHV and post-PHV for FMI (3.94 ± 0.68 vs. 3.29 ± 0.48, *p* = 0.002) and pre-PHV differing from circa-PHV and post-PHV, regarding FFMI values (15.98 ± 2.69 vs. 18.61 ± 2.06 *p* = 0.016; 15.98 ± 2.69 vs. 18.77 ± 2.19, *p* = 0.008, respectively).

### 3.3. Correlation between Years from Peak Height Velocity (YPHV) and Anthropometric Measures

Correlation analysis showed that YPHV had a significant relationship with weight, height, arm span, sitting height, MAC, MAMC, MAA, MAMA, AFI, WC, W/Hr, %PAH, BMI, FM, %FM, and FFMI. Table 2 contains the complete panel of correlations.

Specifically, height (r = 0.921), sitting height (r = 0.941), and %PAH (r*_s_* = 0.941) showed a very strong positive correlation. In addition, weight (r = 0.767), arm span (r = 0.875), MAMC (r*_s_* = 0.809) and MAMA (r = 0.763) highlighted a strong direct correlation, while %FM (r*_s_* = −0.878) had strong negative correlation. Also, MAC (r = 0.550), MAA (r = 0.541), WC (r = 0.405), fat mass (r = 0.404), and FFMI (r = 0.457) presented a moderate positive correlation, whereas AFI (r*_s_* = −0.437) showed a moderate negative relationship. Finally, W/Hr (r*_s_* = −0.343) and BMI (r = 0.319) showed weak negative and positive correlations, respectively. 

On the other hand, AS/Hr, SH/Hr, subscapular skinfold, MAFA, PAH, FMI, and triceps skinfold did not show a significant correlation with YPHV, although triceps skinfold was close to statistical significance, presenting a weak negative correlation (r = −0.288, *p* = 0.065).

### 3.4. Categories for Body Mass Index (BMI), Waist Circumference (WC), Waist to Height Ratio (W/Hr), Birth Quartile, and Achievement of Peak Growth

As stated before, the participants were assigned to the three groups representing the different maturity status (pre-PHV, circa-PHV, and post-PHV), based on their YPHV.

**Figure 1:**
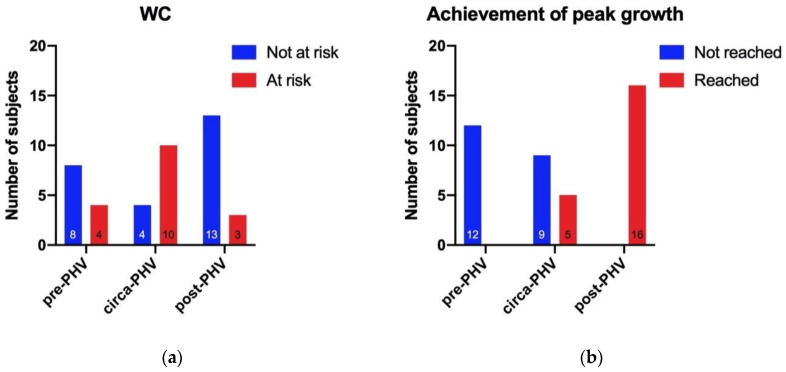

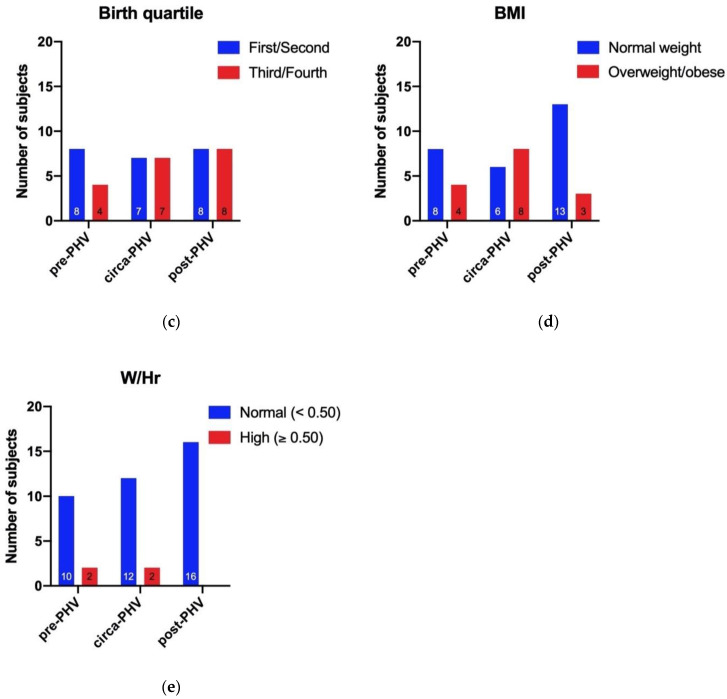
This figure summarizes the association of the three groups of goalkeepers with (**a**) WC, (**b**) achievement of peak growth, (**c**) birth quartile, (**d**) BMI, and (**e**) W/Hr. BMI = body mass index; WC = waist circumference; W/Hr = waist to height ratio.

When the data met the required conditions, the χ^2^ test of independence was used to check if the association between the groups of goalkeepers and the selected categories were present. Otherwise, the number of subjects and frequencies were calculated (Figure 1).

The selected categories were: BMI (divided into “normal weight” and “overweight/obese”), WC (divided into “not at risk” and “at risk”), birth quartile (divided into “first/second” and “third/fourth”), W/Hr (divided in “normal” and “high”), and achievement in peak growth (divided in “reached” and “not reached”).

An association between maturity status and WC categories (χ^2^ = 8.956, *p* = 0.011) was seen (Figure 1a). The circa-PHV group showed the highest number of subjects with a WC >90th percentile and thus was considered at risk regarding this factor (i.e., 10 subjects, representing 71.43% of the circa-PHV group). In the pre-PHV group, 4 subjects (33.33%) were considered at risk, and in the post-PHV, 3 goalkeepers (18.75%) were in the same condition. Taking into account the entire sample of goalkeepers, 17 of them (40.48% of the total) had a WC >90th percentile, while 25 (59.52% of the total) had a normal WC.

Moreover, as expected, a strong association between maturity status and peak growth achievement was seen (Figure 1b; χ^2^ = 29.143, *p* = <0.001). Indeed, all the participants appertaining at the pre-PHV group (*n* = 12, 100.00%) had not reached the peak growth, whereas all the subjects in the post-PHV group had reached the peak growth (*n* = 16, 100.00%). Regarding the circa-PHV, five subjects (35.71%) had not reached the peak growth, while 9 of them (64.29%) had reached it. Hence, half of the goalkeepers (*n* = 21, 50.00% of the total) had reached the peak growth, and the other half (*n* = 21, 50.00% of the total) had not reached it.

Regarding the birth quartile, the χ^2^ test did not showed significance (Figure 1c; X^2^ = 0.961, *p* = 0.618). The number of subjects born in the first/second quartiles of the year were distributed as follows: pre-PHV, *n* = 8, 66.67%; circa-PHV, *n* = 7, 50.00%; post-PHV, *n* = 8, 50.00%. Overall, 23 participants (54.76%) were born in the first half of the year, while 19 (45.24%) in the second. 

BMI and W/Hr data did not meet the required criteria to run the χ^2^ test (Figure 1d,e respectively). However, in the pre-PHV group, 4 subjects (33.33%) were overweight/obese, while in the circa-PHV group, 8 subjects (57.14%), and in the post-PHV group 3 (18.75%) subjects were overweight/obese as well. Overall, on the 42 participants, 15 (35.71% of the total) were in overweight/obese condition, and 27 (64.29% of the total) were normal weight.

Finally, two subjects in the pre-PHV group (16.67%) and two subjects in the circa-PHV group (14.29%) presented a high W/Hr.

## 4. Discussion

In the present study, we investigated the influence of maturity status on the anthropometric profile and body composition of youth goalkeepers from different soccer teams, dividing them considering their somatic maturation state according to their YPHV. In addition, we evaluated whether a RAE was present in the different groups of goalkeepers. 

The main results were that, although participating in sport, the group of youth goalkeepers included a high percentage of subjects with unhealthy body composition and that the worst condition was found for the circa-PHV group. Secondly, regarding the RAE, participants in the first and second half of the year were almost equally distributed, and no association was found regarding the number of goalkeepers of the first/second birth quartile and state of maturity. This result agrees with literature reporting that selection based on maturity is more evident in high-level teams [18]. On the other hand, despite the lack of association between goalkeepers’ birth quartile and state of maturity, the circa-PHV group was composed mainly of participants who reached the peak growth (64.29%). This result suggests that it is important to pay particular attention to the state of maturity when children are very close to the growth spurt.

As expected, the state of maturity status affected the anthropometric profile and body composition of the goalkeepers. The statistical analysis showed differences in most of the measured variables, indicating an adverse condition regarding body composition for the goalkeepers, mainly in the circa-PHV group compared to the post-PHV group. 

For example, although there were differences in age and maturity state, the weight and BMI were similar between circa-PHV and post-PHV, indicating a possible excess in body fat in the younger participants. This aspect was confirmed by comparing body skinfolds and derived body fat measures such as %FM and FMI. We found that triceps skinfold was higher in the circa-PHV, as well as %FM and FMI (Table 1). Although %FM was higher in pre-PHV compared to the others, when fat mass was normalized for height, the circa-PHV group emerged as that with the poorest body composition. Furthermore, the subscapular skinfold was similar among the three groups of goalkeepers but also, in this case, the higher value was found in the circa-PHV. 

Considering the entire sample of goalkeepers, 17 (40.48% of the total) showed a high WC that indicated abdominal obesity and increased cardiometabolic risk. A higher number of subjects with abdominal obesity were in the circa-PHV group. Moreover, W/Hr was different between the three states of maturity, and both the pre-PHV and the circa-PHV presented two goalkeepers having the W/Hr higher than 0.5. Waist circumference shows a high correlation with visceral adipose tissue, plasmatic level of lipids, lipoproteins, and the hormone insulin [34], and W/Hr represents a predictor of cardiovascular risk in children and adolescents [28,34].

Assessing anthropometric variables and body composition close to puberty is crucial because it could also predict adult body composition and future health [44]. Typically, in males, there is an increase in FM at 8 years to 14 years, then FM tends to decrease at about 16 years and subsequently tends to reach a plateau [45]. According to this evidence, the subjects appertaining at the circa-PHV group showed increasing adiposity values in our study. However, a major part of them showed pathological conditions and anthropometric values that place them at risk for cardiovascular and metabolic diseases. Furthermore, it is demonstrated that goalkeepers tend to be heavier, taller, and have larger skinfolds than outfielders [13]. However, although it seems that during puberty, FM typically increases and that goalkeepers present higher anthropometric values, in this study, such conditions are exacerbated. Indeed, factors other than normal growth trends could cause such non-optimal condition for circa-PHV.

Overall, an important consideration is that many of the examined youth goalkeepers were in a condition of overweight/obesity and/or presented high WC and W/Hr. In recent years, a worrying increase in pediatric adiposity was observed almost worldwide [46]. Several non-heritable factors contributed to this obesity pandemic among children and adolescents, including nutrition, physical activity, sports participation, sedentary behavior and electronic devices usage, and parental modeling [46,47,48,49,50]. In the present study, a large proportion of participants showed an unfavorable body composition, despite sport participation. In this regard, it is well known that physical exercise and training are potent stimuli that elicit positive adaptation [51,52,53,54,55,56,57,58,59,60]. However, a previous study indicated that youth soccer players might fail to reach the recommended physical activity levels during days without sports practice. Furthermore, the less active they were off-training, the less they moved during training practice [21]. Thus, such off-training behaviors could be a major cause of the condition found in our sample [61,62].

In addition, goalkeepers tend to have a lower energy expenditure than outfielders, and such a situation is also reported for high-level professional athletes [63]. Thus, more attention should be paid to youth goalkeepers playing in lower-level teams.

Finally, anthropometry is a valuable tool for monitoring harmful situations associated with adiposity, hormonal status [37], and the extent of cardiovascular risk among children and adolescents [38], as well as avoiding early selection.

### Limitations

In this study, we do not measure the levels of physical activity of the participants. However, such an assessment could be useful in future studies investigating anthropometric profile and body composition in youth populations.

## 5. Conclusions

In conclusion, this study evaluates the anthropometric profile trend of a group of goalkeepers, taking into account the state of maturity. The majority of the variables followed the physiological trend occurring during puberty. However, among the entire examined group, a non-optimal anthropometric situation for many subjects indicated an increased risk of cardiometabolic disease. The results of this study add shreds of evidence that also youth goalkeepers participating in sport could be at health risk, and thus attention should be made to factors known to affect body composition and health (e.g., physical activity, sedentary time, and nutrition habits). Thus, monitoring the anthropometric profile and body composition of children and adolescents participating in sport should be an essential aspect to consider in evaluating possible risk conditions and adopting effective countermeasures.

## Figures and Tables

**Table 1:** Descriptive data of goalkeepers based on maturity status.

	Pre-PHV (*n* = 12)		Circa-PHV (*n* = 14)		Post-PHV (*n* = 16)			
	*M ±* *SD*	Min–Max	*M ±* *SD*	Min–Max	*M ±* *SD*	Min–Max	*p*	*η^2^_p_*
**YPHV ^a,b,c^**	−2.02 ± 0.93	−4.10–−1.1	−0.26 ± 0.74	−1.0–1.0	1.99 ± 0.71	1.10–3.60	<0.001	0.822
**Age (years) ^a,b,c^**	11.43 ± 1.37	8.80–13.04	12.86 ± 1.33	10.35–15.0	15.58 ± 0.62	14.30–16.70	<0.001 †	-
**Height (cm) ^a,b,c^**	149.85 ± 7.64	133.30–157.70	166.43 ± 7.61	156.65–179.35	177.00 ± 5.22	171.30–189.30	<0.001	0.737
**Weight (kg) ^a,b^**	44.06 ± 7.88	25.90–54.70	62.66 ± 10.17	47.30–84.55	69.29 ± 10.51	57.80–99.00	<0.001	0.551
**Sitting Height (cm) ^a,b,c^**	77.23 ± 3.55	69.0–81.50	85.94 ± 4.29	82.0–94.0	92.69 ± 3.53	88.0–99.0	<0.001	0.738
**Arm Span (cm) ^a,b,c^**	152.13 ± 10.28	130.60–161.0	169.9 ± 9.11	159.0–185.05	180.13 ± 6.95	169.45–192.0	<0.001	0.640
**AS/Hr**	1.02 ± 0.03	0.95–1.04	1.02 ± 0.02	0.99–1.05	1.01 ± 0.02	0.98–1.04	0.522 †	-
**SH/Hr**	0.52 ± 0.01	0.50–0.54	0.52 ± 0.01	0.48–0.54	0.52 ± 0.01	0.50–0.54	0.330 †	-
**Triceps Skinfold ^c^**	13.77 ± 6.44	5.50–23.0	15.61 ± 5.91	5.50–25.0	9.97 ± 3.31	6.0–18.0	0.042 †	-
**Subscapular Skinfold**	8.31 ± 4.88	3.50–20.75	10.36 ± 4.38	5.0–21.0	7.63 ± 1.99	5.0–12.75	0.143 †	-
**MAC (cm) ^a,b^**	23.68 ± 2.87	17.50–27.50	26.89 ± 4.31	15.90–33.90	28.30 ± 2.03	25.50–34.0	0.002	0.275
**MAMC (cm) ^a,b,c^**	19.36 ± 1.59	15.62–22.12	21.99 ± 3.70	11.19–26.05	25.17 ± 1.57	22.63–28.35	<0.001†	-
**MAA (cm^2^) ^a,b^**	45.26 ± 10.51	24.38–60.21	58.96 ± 17.20	20.13–91.50	64.07 ± 9.54	51.77–92.04	0.002	0.281
**MAMA (cm^2^) ^a,b,c^**	30.02 ± 4.77	19.42–38.94	39.52 ± 11.17	9.97–54.03	50.63 ± 6.39	40.78–63.98	<0.001	0.543
**MAFA (cm^2^)**	15.23 ± 7.99	4.97–27.47	19.44 ± 9.03	5.81–37.47	13.45 ± 5.13	7.37–28.06	0.168†	-
**AFI ^b,c^**	31.81 ± 11.26	15.08–45.63	32.79 ± 10.77	15.08–50.47	20.64 ± 5.46	13.00–30.94	0.005†	-
**WC (cm) ^a,b^**	66.59 ± 6.59	54.40–79.50	75.21 ± 6.96	62.0–87.40	74.09 ± 4.47	68.25–87.15	0.001	0.285
**W/Hr**	0.46 ± 0.05	0.39–0.55	0.45 ± 0.04	0.37–0.53	0.42 ± 0.02	0.38–0.48	0.029†	-
**PAH (cm)**	180.98 ± 3.26	175.29–186.42	179.23 ± 7.88	166.90–190.61	182.16 ± 6.07	174.39–197.91	0.434	0.042
**%PAH ^a,b,c^**	82.79 ± 3.76	75.28–86.62	92.89 ± 2.87	86.54–95.60	97.22 ± 2.97	87.74–99.86	<0.001 †	-
**BMI (kg/m^2^) ^a,b^**	19.62 ± 3.38	13.60–26.00	22.57 ± 2.69	18.50–27.60	22.06 ± 2.64	18.90–30.0	0.031	0.163
**FM (kg) ^a,b^**	8.11 ± 1.47	5.27–10.98	10.93 ± 2.22	7.30–14.99	10.33 ± 1.76	8.59–15.50	0.001	0.295
**%FM ^a,b,c^**	18.47 ± 1.25	16.81–20.65	17.36 ± 1.27	15.02–19.47	14.87 ± 0.58	13.86–15.96	<0.001†	-
**FMI ^c^**	3.63 ± 0.75	2.77–5.21	3.94 ± 0.68	2.77–5.37	3.29 ± 0.48	2.64–4.71	0.016†	-
**FFMI ^a,b^**	15.98 ± 2.69	10.85–20.77	18.61 ± 2.06	15.69–22.37	18.77 ± 2.19	16.06–25.33	0.005	0.235

Pre-PHV = pre-peak height velocity; Circa-PHV = circa-peak height velocity; Post-PHV = post-peak height velocity; YPHV = years from peak height velocity; AS/Hr = arm span to height ratio; SH/Hr = sitting height to height ratio; MAC = mid-arm circumference; MAMC = mid-arm muscle circumference; AFI = arm fat index; WC = waist circumference; PAH = predicted adult height; %PAH = percentage of predicted adult height; BMI = body mass index; FM = fat mass; %FM = percentage of fat mass; FMI = fat mass index; FFMI = free fat mass index; W/Hr = waist to height ratio. Data are expressed as mean ± standard deviation. ^a^ indicates statistical significance for pre-PHV vs. circa-PHV; ^b^ indicates statistical significance for pre-PHV vs. post-PHV; ^c^ indicates statistical significance for circa-PHV vs. post-PHV; † indicates Kruskal–Wallis results.

**Table 2:** This table shows the correlation between YPHV and anthropometric measures.

Selected Variables	Correlation with YPHV (Pearson’s r or Spearman’s Rho)	*p*	Lower 95% CI–Upper 95% CI
**Weight**	0.767	<0.001	0.604–0.869
**Height**	0.921	<0.001	0.856–0.957
**Arm Span**	0.875	<0.001	0.778–0.931
**AS/Hr**	−0.138 †	0.382	−0.424–0.173
**Sitting Height**	0.941	<0.001	0.892–0.968
**SH/Hr**	0.289 †	0.064	−0.017–0.545
**Triceps Skinfold**	−0.284 †	0.069	−0.541–0.022
**Subscapular Skinfold**	−0.034 †	0.830	−0.335–0.273
**MAC**	0.550	<0.001	0.295–0.732
**MAMC**	0.809 †	<0.001	0.670–0.893
**MAA**	0.541	<0.001	0.284–0.726
**MAMA**	0.763	<0.001	0.598–0.866
**MAFA**	−0.087 †	0.584	−0.381–0.223
**AFI**	−0.437 †	0.004	−0.654–−0.153
**WC**	0.405	0.008	0.115–0.631
**W/Hr**	−0.343 †	0.026	−0.586–−0.043
**PAH**	0.159	0.313	−0.152–0.442
**%PAH**	0.941 †	<0.001	0.893–0.968
**BMI**	0.319	0.039	0.017–0.568
**FM**	0.404	0.008	0.114–0.631
**%FM**	−0.878 †	<0.001	−0.933–−0.783
**FMI**	−0.260 †	0.097	−0.522–0.048
**FFMI**	0.457	0.002	0.178–0.668

YPHV = years from PHV; AS/Hr = arm span to height ratio; SH/Hr = sitting height to height ratio; MAC = mid-arm circumference; MAMC = mid-arm muscle circumference; AFI = arm fat index; WC = waist circumference; PAH = predicted adult height; %PAH = percentage of predicted adult height; BMI = body mass index; FM = fat mass; %FM = percentage of fat mass; FMI = fat mass index; FFMI = free fat mass index; W/Hr = waist to height ratio; † indicates Spearman’s Rho.
